# Effect of Brazilian red propolis on microtensile bond strength and sealing ability of the methacrylate-based bonding agents

**DOI:** 10.4317/jced.62587

**Published:** 2025-06-01

**Authors:** Gustavo César Feitosa Vidal, Edson Philippe Bezerra Balbino, José Marcos dos Santos Oliveira, Natanael Barbosa dos Santos, Teresa de Lisieux Guedes Ferreira Lôbo, Ticiano Gomes do Nascimento, Johnnatan Duarte de Freitas, Jeniffer Mclaine Duarte de Freitas, Isabel Cristina Celerino de Moraes Porto

**Affiliations:** 1 Federal University of Alagoas, Faculty of Dentistry; 2 Federal University of Alagoas, Institute of Pharmaceutical Sciences; 3 Federal Institute of Alagoas

## Abstract

**Background:**

The aim of the study was to analyze the sealing ability and microtensile bond strength of a dental adhesive with propolis.

**Material and Methods:**

Red propolis extract was added to an experimental methacrylate-based adhesive (EA) at following concentrations: 100 (EA PV100), 250 (EA PV250) e 500 μg/mL (EA PV500). Single Bond Universal adhesive (SB) and EA without propolis were used as controls. The effectiveness of the dentin sealing was evaluated at nanoscale (silver uptake) and microscale (dye microleakage). Microtensile bond strength (µTBS) was measured 24 hours after the bonding process. Data were analyzed by one-way ANOVA and Tukey’s test (α=0.05).

**Results:**

EA PV500 showed the best coronal sealing, with better performance than the control and the commercial adhesive. Immediate µTBS values ranged from 28.94 + 9.25 MPa (EA PV100) to 39.09 + 9.60 MPa (SB). Comparing SB and EA with propolis, a significant decrease in µTBS was noted (*p*<0.05). The EA without propolis had similar performance to SB (*p*=1.000).

**Conclusions:**

Nanoleakage and microleakage were not eliminated in any of the groups, but EA PV500 µg/mL exhibited the best sealing ability, with superior performance compared to the control and commercial adhesive. A decrease in μTBS of AE with propolis was observed.

** Key words:**Dentin-bonding agents, Propolis, Dental materials, Dental marginal adaptation.

## Introduction

An ideal adhesive filling must have a strong and long-lasting bonding at the interface between material and both enamel and dentin. These are the primary dental substrates which are bonded restorative materials such as composite resin. However, simultaneously bonding the restorative material to two conflicting dental substrates represents a significant challenge in adhesive dentistry. The micromechanical interaction of adhesives with enamel results from the diffusion and entrapment of resin monomers in the microporosities created by the acidic chemical dissolution of enamel. In contrast to enamel, dentin is a moist substrate, with a higher proportion of organic material and permeated by dentinal fluid, making dentin inherently moist throughout its structure. Dentin adhesion has been one of the most challenging and unpredictable tasks in adhesive dentistry due to the dynamic differences in composition and complex histology of dentin. The ideal adhesion ability of restorative materials to dentin is affected by various factors, including biological and clinical factors. These factors encompass patient age, tooth location in the mouth, depth and permeability of dentin, pulp fluid flow, presence of sclerotic and/or carious dentin, root versus coronal dentin, type of restorative material and its application, field isolation, dentist’s experience, among others (1).

Composite resins are the most popular materials for restoring caries lesions due to their good aesthetics and the possibility of minimally invasive treatment. However, to bond them to dental tissues, the use of adhesives is necessary (2).

The bond between the composite and the dental structure must ensure marginal integrity and restoration retention during functional loads, thermal stress, water sorption, and dimensional changes (3). A common issue in composite resin restorations is the occurrence of microleakage, especially in areas where the resin is bonded to dentin margins.

Resin adhesives used in Dentistry must promote a perfect seal of the cavity by bonding to mineralized dental tissues, thereby reducing marginal microleakage. Microleakage is defined as a clinically undetectable passage of bacteria, fluids, molecules, or ions, by gaps between the cavity wall and the restorative material (1,4,5). The interaction of these factors can accelerate failures and degradation of adhesive bonding, resulting in reduced restoration durability (2).

Many approaches during adhesive restorative procedures have been introduced to reduce microleakage, such as the application of different curing strategies, reduction of polymerization contraction force through incremental layering technique, use of low-viscosity composites, surface treatment, and development of new adhesive systems (6-11). Improvements in bonding agents can contribute to a perfect marginal seal and fewer failures at cavity margins (12), increasing the durability of adhesive restorations in the mouth.

Propolis is a natural resinous material produced by Apis mellifera bees, with a complex composition depending on the available plant sources to bees, exhibiting various biological activities. Many studies have shown that propolis has anti-inflammatory and antimicrobial action, stimulates reparative dentin formation, reduces dentin permeability, is active in the treatment and control of caries, accelerates healing of oral tissues, and attenuates pulp inflammation, which can be beneficial for the durability of adhesive bonding (11,13-16).

Failures in the dentin interface sealing is a challenge for the longevity of restorations (17). The durability of the bond to dentin depends on the effective penetration of monomers into the collagen mesh and on the hermetic sealing of the cavity. The ideal adhesive restorative material must be resistant to hydrolytic degradation, inhibit the action of proteolytic enzymes, exhibit good mechanical strength, be biocompatible, and have antimicrobial and remineralizing action. However, despite significant developments in dental adhesives. But, until now there is no material on the market with all those characteristics (18).

Dental adhesives are placed in close contact with dentin and enamel, acting as a connection between the restorative material and dental tissue and, therefore, have received special attention from researchers to improve the strength and durability of bonding at the adhesive interface. Tests with adhesives containing propolis could add knowledge about the performance of this new material, which will be important to support future studies with this material.

The aim of this study was to analyze the dentin and coronal sealing ability and the adhesive strength of a new dental adhesive with propolis.

## Material and Methods

1. Propolis Extract Obtaining

Raw red propolis was collected in the mangroves of the State of Alagoas, Brazil. Access and transportation of propolis were previously authorized by regulatory agency for control of Brazilian Genetic Heritage and Biodiversity Conservation (protocol number #A88DA2B). The extraction of Alagoas red propolis (PVA) was carried out by maceration in ethanol/water solution (80/20 v.v.) to get the ethanolic extract of raw red propolis (EPVA). Liquid-liquid extraction of the raw extract was performed to eliminate greases and waxes, and the ethyl acetate extract enriched with PVA flavonoids and isoflavonoids was obtained, as described by Porto *et al*. (11). It was used in all experiments in this study.

2. Experimental Adhesives Preparation

A two-step experimental adhesive (EA) was prepared by mixing hydroxyethyl methacrylate (HEMA) and 2,2-bis[4-(2-hydroxy-3-methacryloxypropoxy)phenyl]-propane (BisGMA) monomers with a mass ratio of 40/60. Ethanol/water (80/20 v/v) was used as solvent at a concentration of 3% (w/w), with a two-component photoinitiator system: camphorquinone (0.5%, w/w) and ethyl (4-dimethylaminobenzoate) (0.5%, w/w). Monomers and solvent were mixed in light-protected tubes and then heated (68°C) and homogenized in a shaker, alternating heating and stirring. The initiators were added to the mixture, homogenized at room temperature (22°C). To this adhesive, EAEPV and a mixture of silanized silica (SiO2) and titanium dioxide (TiO2) nanoparticles (1% 50/50 w/w) were added, resulting in adhesives with EAEPV concentrations of 100 µg/mL, 250 µg/mL, 500 µg/mL, and 1% of inorganic fillers. Experimental adhesive without propolis and a commercial adhesive (Single Bond Universal 3M/ESPE, St. Paul, MN, USA) were used as controls. A light-emitting diode source (Emitter B; Schuster Com. Equip. Odontológicos Ltda, Santa Maria, Brazil - 1135 mW/cm2) was used for adhesive and composite polymerization.

3. Teeth Preparation

Thirty-three healthy bovine incisors were used. Teeth were cleaned with periodontal curettes and polished with a paste of pumice stone and water applied with rubber cup attached to a low-speed piece. The root and pulp were removed, and the teeth were stored at 8°C. The sampling was of the stratified random type. For the formation of strata (groups), teeth were numbered, randomly selected, and allocated into groups using PS-Sort software - Porto Sample Sort (Porto-Filho CAB, Brazil), v.2.

4. Dentin/Resin Microtensile Bond Strength

The teeth used for microtensile tests was randomly distributed into the following groups: G1: AE with PV 100 µg/mL (AE PV100), G2: AE with PV 250 µg/mL (AE PV250), G3: AE with PV 500 µg/mL (AE PV500), G4: AE without propolis and without filler (AE), G5: Single Bond 2 (SB), G6: AE without propolis and with filler (AE Np). The incisal third of the teeth was removed, and the pulp chamber was filled with composite resin after dentin acid etching. Then, the enamel of the vestibular surface was removed, and a standard smear layer was created on exposed dentin with 600-grit silicon carbide paper for 60s, rotating ¼ turn every 15s under water irrigation. Adhesives were applied to the acid-conditioned dentin for 15 seconds, then a light air blow was applied for 5s to evaporate the residual solvent, and the adhesive was light-cured for 10 seconds. The composite resin (Z 250XT 3M/ESPE, St. Paul, MN, USA. Color A2) was then applied in 2 increments of 2 mm, light-cured for 20 seconds each. After 24 hours in distilled water at 37°C, the teeth were longitudinally sectioned in the x and y-axis directions to obtain sticks with a cross-sectional area of 1.0 ± 0.1 mm2. The microtensile bond strength test was performed on a semi-universal machine (ODEME, Brazil) at a speed of 0.5 mm/min until failure. The specimens were dentin/resin sticks (n=30 per group). No sample calculation was used, as the methodologies employed in the scientific literature for similar studies were considered, as indicated in scientific publications in the field. This quantity provides data dispersion around the mean and allows the study to draw conclusions relevant to the researched issue. The fracture mode was evaluated at 60X magnification and categorized into four groups: cohesive dentin fracture (DF); cohesive composite resin fracture (RF); adhesive fracture (AF) when failure occurred at the dentin/adhesive interface, or mixed fracture (MF) when two or more fracture modes occurred simultaneously.

5. Dentin Sealing Analysis (nanoleakage)

Two sticks per group prepared for microtensile testing were covered with two layers of nail varnish leaving 1 mm exposed beyond the bonding interface. Then, the samples were immersed in deionized water for 30 minutes before immersion in a 50% ammoniacal silver nitrate (AgNO3) solution for 24 hours in the dark. The ammoniacal silver nitrate solution was prepared by dissolving 25 g of silver nitrate crystals in 25 mL of deionized water. Then, 28% ammonium hydroxide was dripped onto the silver nitrate solution to titrate the solution, which initially became transparent, then darkened, and finally became transparent again, due to the transformation of ammonium ions into diamine silver ions. The resulting solution was diluted in deionized water to 50 mL of the final solution, to obtain a 50% ammoniacal silver nitrate concentration at pH 9.5. After 24 hours in the ammoniacal silver solution, the samples were washed with distilled water and immersed in a developing chemical solution (Carestream Health, Rochester, NY, USA) for 8 hours under fluorescent light. The samples were embedded in epoxy resin and polished with SiC sandpaper (600, 800, 1000, and 1200 Grit) and diamond pastes (3, 1, and 0.25 μm), then dehydrated, gold-coated, and analyzed in SEM in transmitted electron mode at 20 kV.

6. Coronary Sealing Analysis (microleakage)

Fifteen healthy bovine incisors were used, randomly distributed into groups (n=3) as described for the microtensile test. A standardized Class V cavity was made on the vestibular surface of the teeth with a rounded-end cylindrical diamond bur, accoupled to a high-speed piece (# 1141, KG Sorensen, Brazil) under constant irrigation. All preparations were defined with a depth of 2.0 mm, and 4 x 4 mm shape positioned 2 mm above the amelocemental junction (ACJ). The preparations were made by a single trained individual, using a standardized cavity preparation machine (APC 100, Odeme Dental Research, SC, Brazil), and the burs were replaced every 5 cavity preparations. Preparations with pulp exposure were excluded. Dentin was conditioned with 37% phosphoric acid for 15 s, rinsed with water, and excess water removed. Then, the adhesive was applied followed by a light air blow and photoactivation for 10 s. Restoration with Z250XT resin (3M/ESPE, MN, USA. Color A2) was placed and each increment was light-cured for 20 s. After 24 hours in distilled water, 37 oC, the restoration was polished with polishing disks (TDV, Pomerode, BR. Medium, fine, and ultra-fine grit). The restored teeth were sealed with nail enamel, leaving 1 mm free around the restoration margins, then immersed in 2% Rhodamine B, pH 7.0, for 24 hours. Next, they were rinsed under running water for 15 min and dried with absorbent paper before being sectioned in the mesio-distal direction, producing 6 samples per group. The degree of infiltration was assessed under a stereoscopic microscope 60x by three calibrated examiners (Kappa test), considering the most severe degree of infiltration found among the following scores: 0- No dye penetration; 1- Dye penetration at the tooth/restoration interface up to one-third beyond the cavity wall; 2- Dye penetration at the tooth/restoration interface beyond one-third and less than two-thirds beyond the cavity wall; - Dye penetration at the tooth/restoration interface beyond two-thirds or the entire extent of the cavity wall; 4- Dye penetration in the axial wall.

7. Statistical Analysis

Weighted Kappa test was performed to assess the degree of agreement between examiners of coronary microleakage. Dentin bond strength and coronary microleakage data were subjected to one-way ANOVA followed by Tukey’s pairwise comparison test (Statistical Package for the Social Sciences (SPSS), version 26). The error margin used in the statistical test decisions was 5%.

## Results

1. Microtensile Bond Strength

**Figure 1:**
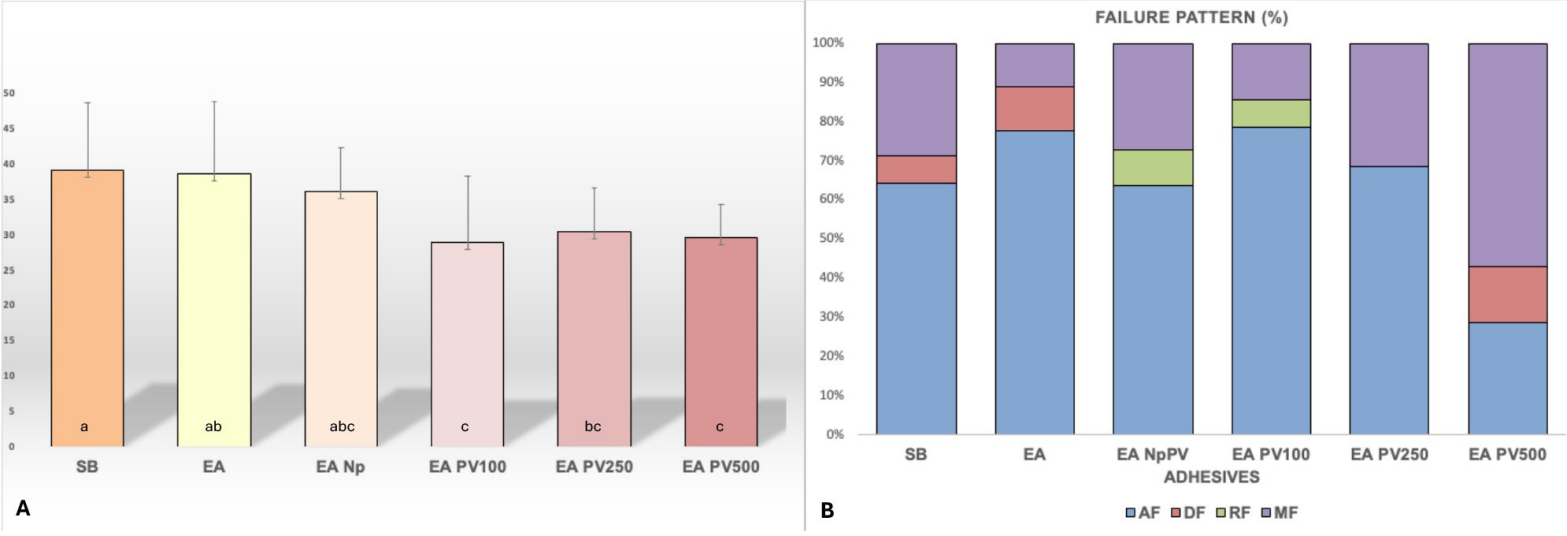
Mean values of microtensile bond strength (MPa) 24h after light-cure (A). SB: Single Bond 2; EA: Experimental unfilled adhesive and without propolis; EA Np: Experimental filled adhesive without propolis; EA PV100: Experimental filled adhesive with 100 ug/mL of propolis; EA PV250: Experimental filled adhesive with 250 ug/mL of propolis; EA PV500: Experimental filled adhesive with 500 ug/mL of propolis. Different lowercase letters indicate significant statistical difference (*p* <0.05). (B): Distribution of fracture pattern (%) in each experimental group. (AF) adhesive failure when failure occurred at the dentin/adhesive interface; (DF) cohesive failure in dentin, when located exclusively within dentin; (RF) cohesive failure in resin, when located exclusively within resin; (MF) mixed failure when more than one mode of failure occurred simultaneously. SB: Single Bond 2; EA: Experimental unfilled adhesive and without propolis; EA Np: Experimental filled adhesive without propolis; EA PV100: Experimental filled adhesive with 100 ug/mL of propolis; EA PV250: Experimental filled adhesive with 250 ug/mL of propolis; EA PV500: Experimental filled adhesive with 500 ug/mL of propolis.

**Figure 2:**
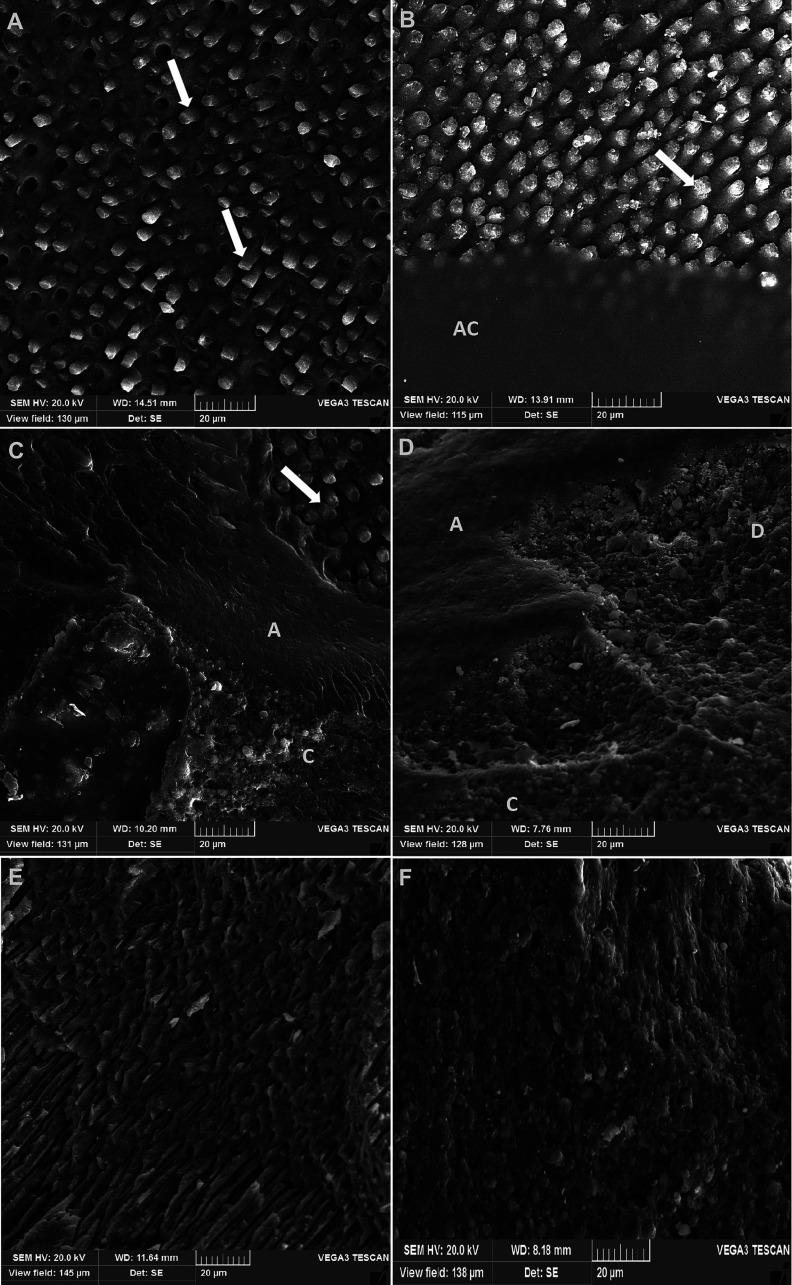
SEM photomicrographs of the failure modes of the microtensile test. A and B: Adhesive failure when failure occurred at the dentin/adhesive interface. Image A shows adhesive failure on bottom of hybrid layer exposing resin tags (white arrows). Image B presents a mixed adhesive failure with broken resin tags (white arrow) and adhesive layer covering (AC) the dentin. C and D: Mixed failure when more than one mode of failure occurred simultaneously. On left side: A-adhesive and C-composite; on right side: A-adhesive, C-composite and D-dentin. E: cohesive failure in resin, when located exclusively within resin. F: cohesive failure in dentin, when located exclusively within dentin.

Figure 1A shows immediate bond strength (24h). Compared to commercial adhesive, the EA with propolis showed a significant reduction in bond strength (*p* < 0.05). The experimental adhesive without propolis performed similarly to the commercial adhesive (*p* > 0.05). The addition of silica and titanium nanoparticles did not disturb the immediate bond strength of the dentin/resin interface. The predominant fracture type for all groups was adhesive fracture (above the hybrid layer), except for AE PV500 group, which showed mostly mixed fracture (Fig. 1B). Figure 2 shows the criteria used in the fracture mode assessment.

2. Nanoleakage

**Figure 3:**
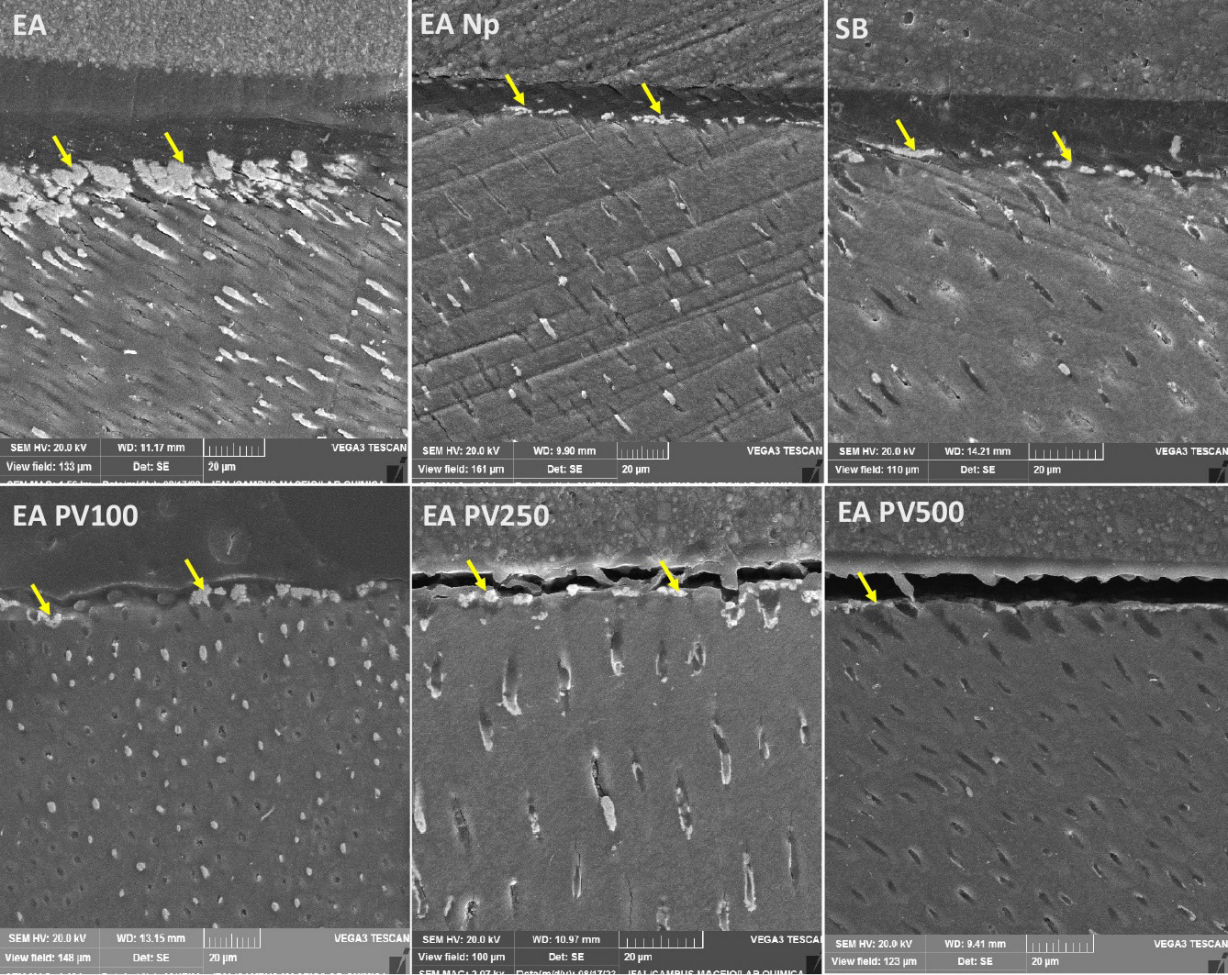
SEM images of nanoleakage expression from different groups. Nanoleakage (yellow narrows) was observed in the base of hybrid layer. SB: Single Bond 2; EA: Experimental unfilled adhesive and without propolis; EA Np: Experimental filled adhesive without propolis; EA PV100: Experimental filled adhesive with 100 ug/mL of propolis; EA PV250: Experimental filled adhesive with 250 ug/mL of propolis; EA PV500: Experimental filled adhesive with 500 ug/mL of propolis.

Typical nanoleakage patterns at the resin-dentin interfaces for each dentin adhesive system are illustrated in Figure 3. AEPV 500 adhesive clearly showed a lower amount of silver particle diffusion in the adhesive interface than other experimental adhesives and control. AEPV 100 and AE PV250 adhesives exhibited larger silver uptake distributed under the hybrid layer and numerous dentin tubules with silver deposition between the resin tags and tubule walls.

3. Coronary Sealing Analysis (Microleakage)

**Figure 4:**
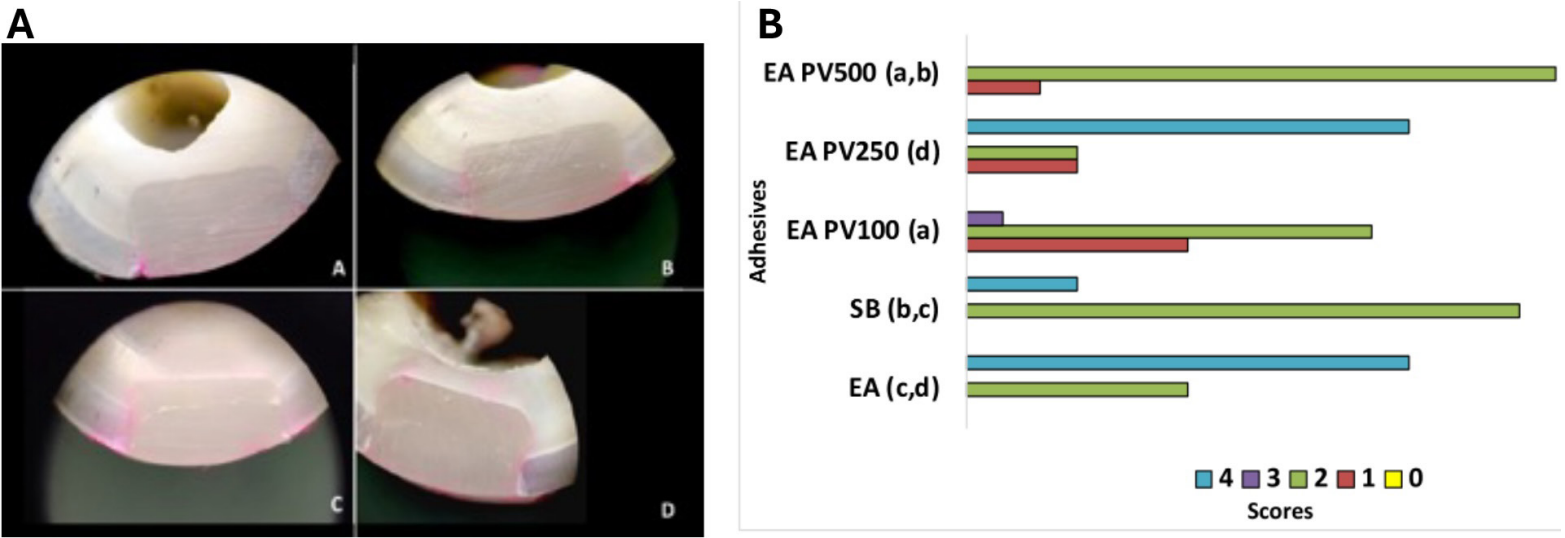
A: Stereomicroscope photographs show dye penetration scores used for microleakage assessment. (60x). No Score 0 (No dye penetration) was recorded. A: Dye penetration at the tooth/restoration interface up to one-third beyond the cavity wall (score 1); B: Dye penetration at the tooth/restoration interface beyond one-third and less than two-thirds beyond the cavity wall (score 2); C: Dye penetration at the tooth/restoration interface beyond two-thirds or the entire extent of the cavity wall (score 3); D: Dye penetration in the axial wall (score 4). B: Coronary sealing ability of the different adhesives tested.0: No dye penetration. 1: Dye penetration at the tooth/restoration interface up to one-third beyond the cavity wall; 2: Dye penetration at the tooth/restoration interface beyond one-third and less than two-thirds beyond the cavity wall; 3: Dye penetration at the tooth/restoration interface beyond two-thirds or the entire extent of the cavity wall; 4: Dye penetration in the axial wall. SB: Single Bond 2; EA: Experimental unfilled adhesive and without propolis; EA PV100: Experimental filled adhesive with 100 ug/mL of propolis; EA PV250: Experimental filled adhesive with 250 ug/mL of propolis; EA PV500: Experimental filled adhesive with 500 ug/mL of propolis. Different lowercase letters indicate significant statistical difference (*p* <0.05).

Reliability between examiners was obtained using SPSS statistical software v.26 through weighted Kappa calculation. The weighted Kappa value was 0.95, indicating an almost perfect level of agreement. Microleakage analysis criteria are presented in Figure 4A. In all groups, dye infiltration occurred in the surrounding walls. Figure 4B presents microleakage data after statistical analysis. No statistically significant difference was found between AE and AE PV250 (*p* = 0.387). Among propolis-containing adhesives, the highest level of infiltration was noted in the AE PV250 group, with dye penetration reaching the axial wall in 4/6 analyzed samples. AE PV100 and AE PV500 groups showed better coronary sealing than AE (control) and SB groups (*p* < 0.05).

## Discussion

This is one of the few studies where propolis was added to dental adhesives. Considering the literature and the results of this research, the addition of natural components as propolis to dentin adhesives may have potentially positive effects on the mechanical and adhesive properties of these materials (11). While the search for natural alternatives is trustworthy, it’s also crucial to understand the potential troubles and search for balance and potential benefits of natural components over the maintenance of mechanical properties. This is a challenge to be considered in formulating new dentin adhesives.

Propolis was chosen to be added into dental adhesive due to its potential properties that could be applied to adhesive dentistry, including anti-inflammatory, antioxidant and antimicrobial activities, stimulation of reparative dentin formation, which are relevant to the preservation of the restored tooth.

Some studies have shown significant amounts of phenolic compounds in Brazilian red propolis. Some of these compounds allow its differentiation from other types of propolis and are known as Brazilian red propolis biomarkers (liquiritigenin, isoliquiritigenin, formononetin, biochanin A, genistein, daidzein, luteolin, naringenin, galangin, pinobanksin, pinocembrin, and quercetin) (19,20), and they are associated with antibacterial (13,21), anti-inflammatory (22,23) and antioxidant activities (11,19).

The antioxidant activity of flavonoids depends on their structure and can occur through the following pathways: 1) reactivity as a donor of hydrogen atoms and electrons, 2) stability of the flavonol radical created; 3) reactivity with other antioxidants, 4) ability to chelate transition metals, and 5) solubility and interaction with cell membranes (24). Polyphenols are also skilled of stabilizing the collagen chain, increasing the number of cross-links in collagen fibrils, reducing their biodegradation (25).

The effectiveness of propolis as an anti-inflammatory is related to various fronts of inflammation attenuation, reducing the expression of inflammatory genes and inhibiting the synthesis of pro-inflammatory cytokines and modulation of the immune response (22). Polyphenols block prostaglandin synthesis by inhibiting cyclooxygenase-2 (COX-2). This can be particularly useful in dentistry because excessive COX-2 production induces matrix metalloproteinase (MMP) activation (23).

MMPs are zinc and calcium-dependent endopeptidases that degrade extracellular matrix components and are involved in dental development and the dentin caries process in the initial stages of demineralization. Changes in collagen and non-collagen protein structure may reduce mechanical properties and the ability to remineralize caries-affected dentin. MMPs are also involved in the degradation of the hybrid layer in composite resin restorations (26,27), degrading the exposed collagen not covered by the resinous adhesive. Thus, MMP inhibition is crucial for preserving the integrity of the dentin matrix and the adhesive interface.

Furthermore, propolis also has the ability to stimulate reparative dentin formation, which is beneficial for regenerating dental tissues, especially in very deep cavities. Studies with propolis have shown comparable or superior results to conventional materials such as Dycal and MTA in terms of pulp response, dentin bridge formation, and anti-inflammatory action. Possibly, the anti-inflammatory and healing properties of propolis have contributed to a positive response in reparative dentin formation (28,29).

The understanding that it was necessary to eliminate microbial contamination from a cavity before placing the restoration has been replaced by the current knowledge that maintaining contaminated dentin under restorations is inevitable and not associated with treatment failure. Thus, selective removal of caries in softened dentin in a single session has been recommended for deep carious lesions to preserve dental vitality (30). In this context, propolis stands out for its antimicrobial activity, especially against *S. mutans*, *Lactobacillus*, *E. faecalis*, among other bacteria (13,21).

Therefore, the addition of propolis to dental adhesive was conceived with the aim of increasing dentin matrix strength, promoting tissue regeneration, and reducing MMP action. These properties can play an important role in improving the effectiveness and durability of dental adhesives, contributing to the success of dental restorations (21).

Although the addition of propolis may confer benefits in terms of antioxidant, anti-inflammatory, and antimicrobial properties, it’s important to understand that its interaction with adhesive components may have undesired effects that still need to be elucidated. Each adhesive in combination with additives may present different responses, and more research is needed to optimize the incorporation of propolis or other compounds without compromising the integrity of the adhesive bond.

Lenzi *et al*. (31) showed that, on average, the diameter of dentinal tubules corresponds to 2.4 ± 0.07 μm in the superficial dentin layer, 3.70 ± 0.06 μm in the middle dentin, and 4.28 ± 0.04 μm in deep dentin, near the pulp chamber. In this study, 20 nm-sized load particles were used, compatible with the diameter of dentinal tubules, allowing load particles to flow with the adhesive resin into the tubules.

Adding load to the adhesive increases its viscosity and reduces its flow, producing a thicker hybrid layer that can absorb the resin contraction stress. The addition of filler of 15 and 10 wt. % in dentin adhesive showed improved bond strength and exhibited a suiTable dentin interaction, but the conversion degree was decreased linearly (32).

Filled adhesives have higher bond strength (32,33) and prevent the adhesive from spreading beyond the desired area. However, they should not have a high amount of filler because it would negatively impact the adhesive penetration and could lead to a formation of a weak hybrid layer (34,35). In this study, it is noted that the amount of 1% silica and titanium dioxide nanoparticles (20 nm) was adequate to allow infiltration into the hybrid layer and reinforce the adhesive bond, as the nanoparticle adhesive performed similarly to the control group.

However, there was a higher number of adhesive fractures, predominantly at the top of the hybrid layer, in all tested groups except the AE PV500 group. In propolis-containing adhesives, there was a decrease in the percentage of adhesive fractures inversely proportional to the concentration of propolis in the adhesive. The predominance of adhesive or mixed fractures in a microtensile test on adhesive materials is an important indication of the quality and effectiveness of adhesion between the adhesive material and the substrate. Adhesive fractures indicate low adhesion between materials, while mixed fractures may suggest deficiencies in both adhesion and material strength. Understanding these failure patterns is essential for improving adhesive materials used in various applications, including dentistry, ensuring durable and reliable restorations. Porto *et al*. (11) showed that propolis-containing adhesives build an appropriate hybrid layer, which possibly occurred in this study. However, the bond of the propolis adhesive to restorative resin seems to have been more fragile, facilitating fracture at the adhesive/resin interface.

In this study, SB, AE, and AENp adhesives showed similar bond strength among them and significantly higher bond strength than propolis-containing adhesives. In the literature, the average microtensile bond strength values for the commercial SB adhesive vary from 22.27 MPa36 to 48.05 MPa37. These studies corroborate the results of the experimental propolis adhesives AE PV100 (28.94 MPa), AE PV250 (30.47 MPa), and AE PV500 (29.58 MPa), suggesting that propolis adhesives have the potential to provide effective adhesion.

Nanoleakage is generally considered a relevant guide to estimate dentin sealing capacity and the effectiveness of bonding of an adhesive system. Ideally, adhesive systems are expected to completely reduce dentin permeability. In this study, none of the adhesive system groups were able to completely reduce dentin permeability produced after conditioning, and all groups showed nanoleakage, regardless of the adhesive composition used. The 500 µg/mL propolis adhesive showed the best immediate dentin sealing competence. Meanwhile, adhesives with propolis at concentrations of 100 and 250 µg/mL showed higher silver uptake, indicating less ability for dentin sealing. Nanoleakage can be increased as much as the layer of non-infiltrated collagen fibrils is larger.

Although it has been demonstrated that there is no agreement between marginal leakage and bond strength (38,39), the latter has been systematically used as the preferred method to assess the effectiveness of adhesive system bonding and infer associations with the clinical performance of composite resin restorations.

The longevity of a restoration also depends on the sealing between the material and the cavity walls (40), In this study, the best coronal sealing was provided by propolis-containing adhesives at concentrations of 100 and 500 µg/mL, and the results of the SB, AE, AENp, and Np250 groups were similar. Although all restorations were made by a single individual, some technical variation may have occurred. Additionally, the polymerization contraction effect and associated stresses generated by the photopolymerization of composite resin may be stronger than bond strength and contribute to the creation of gaps, favoring the presence of adhesive failures and consequently microleakage at the union margin between the tooth and the resin.

The association of marginal gaps and subsequent microleakage with the onset of secondary caries has been frequently reported (41,42). Therefore, microleakage tests are employed to evaluate restoration effectiveness. There are different methods to evaluate microleakage, such as dye penetration, radioactive isotope infiltration, dye extraction, and bacterial culture (43).

Microleakage evaluation with dye penetration has the advantage of being cost-effective, and direct observation allows assessing the depth of infiltration under the microscope (44). The most widely used dyes are methylene blue and rhodamine B. However, attention should be paid to the choice of the dye used. The lower molecular weight (319.85 g.mol-1) and smaller diameter than most bacterial cells make methylene blue the preferred dye (45). However, the smaller size of methylene blue molecules (molecular area of 0.52 nm²) can lead to overestimation of microleakage (43). In this study, we used Rhodamine B 2% (molecular weight 479.02 g.mol-1) to evaluate microleakage around class V restorations due to its small particle size (molecular area of 1.75 nm²), suiTable penetration, good water solubility, spreading, and non-reactivity with hard tissues (46,47).

Microleakage is the permeability in the space between the restorative material and the cavity walls, allowing infiltration of microorganisms, molecules, and ions, which can cause recurrent caries, pulp inflammation, and marginal discoloration. It can be caused by fluctuations in the physical structures involved (tooth/restorative material), among others, the polymerization contraction of the composite resin, the thermal expansion coefficient, and the modulus of elasticity (48).

In this study, a conventional experimental adhesive was developed, requiring acid conditioning of enamel and dentin. Adhesives applied through total acid conditioning of enamel and dentin have higher microleakage than self-conditioning systems (47,49). Additionally, according to Jain *et al*. (50), other factors that can influence microleakage in teeth are the C-factor, enamel bevel failure, and the composition and structure of dental dentin in class V cavities. In this study, cavity preparation was standardized, and during the restorative procedure, the gradual photoactivation technique and resin insertion in increments were employed to optimize restoration. Furthermore, diamond tips were replaced every five cavity preparations. Emir *et al*. (51) and Porto *et al*. (52) showed that after the fifth cavity preparation, diamond tips lose their cutting edge due to wear and/or loss of diamond particles, making the surface less rough.

This was an *in vitro* study to evaluate the bond strength and sealing ability of a new dental adhesive. Laboratory studies are essential in dental research, marking the development and scientific knowledge of dental materials, bioactive substances, among others. However, any laboratory advance must be validated in a clinical setting since many other variables are involved and can contribute synergistically to the longevity of the restoration.

The bond strength to dentin of the adhesives was reduced by the addition of propolis. Nanoleakage was not eliminated in any of the groups, but the adhesives with 500 µg/mL propolis exhibited the best coronal sealing, with superior performance compared to the control and commercial adhesive.

## Data Availability

The datasets used and/or analyzed during the current study are available from the corresponding author.
